# What professional activities do general practitioners find most meaningful? Cross sectional survey of Norwegian general practitioners

**DOI:** 10.1186/1471-2296-14-41

**Published:** 2013-03-23

**Authors:** Peder Andreas Halvorsen, Adrian Edwards, Ivar Johannes Aaraas, Olaf Gjerløw Aasland, Ivar Sønbø Kristiansen

**Affiliations:** 1 National Centre of Rural Medicine, Department of Community Medicine, University of Tromsø, Tromsø N-9037, Norway; 2 General Practice Research Unit, Department of Community Medicine, University of Tromsø, Tromsø N-9037, Norway; 3 Cochrane Institute of Primary Care and Public Health, School of Medicine, Cardiff University, Neuadd Meirionnydd, Heath Park, Cardiff, CF14 4XN, UK; 4 The Research Institute, The Norwegian Medical Association, P. box 1152 Sentrum, Oslo, N-0107, Norway; 5 Department of Health Management and Health Economics, Institute of Health and Society, University of Oslo, P. box 1089, Blindern, Oslo, N-0318, Norway; 6 Peder A. Halvorsen, Svartaksveien 15, Alta, N 9516, Norway

**Keywords:** Health priorities, Health care reforms, General practice

## Abstract

**Background:**

Health reforms in many countries affect the scope and nature of primary care. General Practitioners (GPs) are expected to spend more time developing public health, preventive health care, coordination of care and teamwork. We aimed to explore which professional activities GPs consider to be meaningful and how they would like to prioritise tasks.

**Methods:**

In a cross sectional online survey 3,270 GPs were invited to consider twenty different activities in general practice. They were asked to rate each of them on a Likert scale anchored from 1 (not meaningful) to 5 (very meaningful). They then selected three activities from the item list on which they would like to spend more time and three activities on which they would like to spend less time. We used multinomial logistic regression to explore associations between the GPs’ preferences for time spent on preventive health care activities and age, gender and practice characteristics.

**Results:**

Approximately 40% (n=1,308) responded. The most meaningful activities were handling common symptoms and complaints (94% scored 4 or 5), chronic somatic diseases (93%), terminal care (80%), chronic psychiatric diseases (77%), risk conditions (76%) and on call emergency services (70%). In terms of priority the same items prevailed except that GPs would like to spend less time on emergency services. Items with low priority were health certificates, practice administration, meetings with local health authorities, medically unexplained symptoms, addiction medicine, follow up of people certified unfit for work, psychosocial problems, preventive health clinics for children and school health services. In multivariate regression models physician and practice characteristics explained no more than 10% of the variability in the GPs’ preferences for time spent on preventive health care services.

**Conclusions:**

The GPs found diagnosis and treatment of diseases most meaningful. Their priorities were partly at odds with those of the health authorities and policy makers.

## Background

Despite substantial health reforms in many countries affecting the scope and nature of primary care, little is known about the aspects of care that GPs find most meaningful and valuable for themselves and their patients. Strong primary care is frequently seen by policy-makers as essential for effective and cost-effective health care [1,2]. Qualities of primary care include provision of personal and personalised health care, knowledge of patients’ values and preferences, and patient trust that GPs will secure the appropriate care that they need. Many health reforms seek to build on the strengths of their primary health care sectors, but also to contain secondary care costs, improve chronic disease management and increase preventive activities [3-10]. In the UK, GPs are being given unprecedented responsibility for commissioning services at regional level [6]. In Canada and the Netherlands a diverse range of primary health care reform initiatives have been implemented, including the development of primary health care teams and networks, blended payment systems, increasing the workforce and measures to improve quality and safety [7,8]. In Norway the Coordination Reform is currently being implemented. Coordination of care, preventive medicine and cost containment are salient priorities [5].

Primary health care reforms will necessarily involve all kinds of health personnel, but general practitioners (GPs) in particular have been expected to take on new responsibilities [5]. This, however, may come at a cost in terms of money or other activities forgone. If GPs are to use more time on preventive measures and team work, less time will be available for individual consultations with their patients. More GPs may be needed, or else current GPs may have to work more. However, it is not clear to what extent the priorities of policy-makers and the health authorities are consistent or compatible with those of the GPs themselves. If they are incongruent, the aims of primary health care reforms may be difficult to achieve.

It has been shown that Norwegian GPs spend about 70% of their total working hours on direct work with patients [11]. A majority of Norwegian GPs would prefer a shorter working week, but the proportion that perceive their workload as unacceptable is now less than 40% and decreasing [11]. In other countries it has been shown that relationships with patients [12-14], clinical competence [12,15], and clinical autonomy [14] are highly valued by physicians including GPs. Conversely, some studies have identified administrative burdens, paperwork and governmental regulations as sources of dissatisfaction [12,16].

What GPs would like to prioritize for themselves may not necessarily be the same as what is in their patients’ best interest. Nevertheless, for health authorities, policymakers and GP leaders who wish to implement health reforms in patients’ best interests, knowledge about how GPs would like to spend their time seems important. In the present study we aimed to explore what GPs might wish to prioritize among a broad range of common activities in general practice. Specifically, we asked to what extent different work activities were considered meaningful and whether GPs would like to spend more or less time on them.

## Methods

In December 2009 3,270 GPs registered with the Norwegian Medical Association in Norway were sent an e-mail asking them to participate in an online survey pertinent to the forthcoming Coordination Reform [5]. We aimed to include all GPs in Norway at the time (n= 4,049, Table 1), but GPs engaged in another survey taking place at the same time were excluded. Thus a random sample comprising 81% of all Norwegian GPs were invited. The online questionnaire was administered by the Research Institute of the Norwegian Medical Association (NMA). The front screen gave a short presentation of the Coordination Reform and stated that knowledge about GPs’ views about core issues pertaining to the reform was needed. Return of the online, anonymous questionnaire was considered as consent to participate in the study.

**Table 1:** Respondent characteristics

**Variable**	**Respondents**	**All Norwegian GPs**
	**n=1,308**	**n=4,049**^**1**^
Age, mean	47 y	49 y^2^
Females	36%	35%^2^
Specialty attainment	66%	55%^3^
Mean number of patients listed per doctor	1,209	1,182^2^
Municipality, number of inhabitants		
<5,000	13%	14%^1^
5,000-9,999	13%	14%^1^
10,000-19,999	18%	17%^1^
20,000-49,999	24%	21%^1^
50,000 +	32%	34%^1^

1. Statistics Norway (http://www.ssb.no accessed 24th of March 2011).

2. http://www.helsedirektoratet.no/finansiering/refusjonsordninger/tall-og-analyser/Documents/hovedtallsrapport-2010.pdf.

3. http://www.legeforeningen.no/id/18 14.04.2011.

The GPs were presented with a list of twenty items covering a broad range of activities in general practice, such as handling common symptoms and complaints, follow up of chronic diseases, preventive health care, teaching, research and administrative tasks (Table 2). The GPs were asked to rate each item on a Likert scale anchored at 1 (not meaningful) and 5 (very meaningful). Subsequently, they were asked to select three activities from the list – in order of priority - on which they would like to spend more time and three activities on which they would like to spend less time. Additionally, they were asked about preferences for practice organisation and remuneration (reported elsewhere) [17]. During the data collection period these questions raised some discussion and criticism in an internet forum for Norwegian GPs, including doubts about whether the study was independent of governmental reform interests (although it was independent). The main outcome measures were consideration of meaningfulness and priority of job tasks.

**Table 2:** GPs' ratings of meaningfulness of common activities in general practice on a scale anchored from 1 (not meaningful) to 5 (very meaningful)

**Activity**	**Score 4 or 5 (n=1,308)**^**1**^
**Recent everyday symptoms and complaints**	94%
(*e.g. *infections, lumbago, tendinitis, head ache, dyspnea, chest pain, abdominal pain, *etc.*)	
**Follow up of chronic somatic diseases**	93%
(*e.g.* COPD, heart disease, diabetes)	
**Terminal care**	80%
**Follow up of chronic psychiatric diseases**	77%
(*e.g.* schizophrenia, bipolar disorders, anxiety/depression)	
**Risk conditions**	76%
(elevated blood pressure or cholesterol, low bone mass density)	
**On call emergency health care**	70%
(*e.g.* trauma/accidents, acute, serious somatic and psychiatric diseases)	
**Meetings regarding individual patients**	67%
**Teaching and supervision of students and residents**	64%
**Follow up of persons certified unfit for work**	52%
**Psychosocial problems**	49%
(*e.g. *marital crises, conflicts at work)	
**Nursing home medicine**	48%
**Quality assurance**	44%
(*e.g.* development and maintenance of guidelines/procedures)	
**Medically unexplained symptoms**	44%
(*e.g. *chronic fatigue, chronic pain syndroms)	
**Drug abuse/addiction medicine**	41%
**Meetings with local health authorities**	35%
**Preventive health clinics**	32%
**Research**	29%
**Practice administration/management**	29%
(*e.g. *human resource management, bookkeeping, *etc.*)	
**School health service**	21%
**Health certifications**	16%

1) Instead of providing a score the GPs’ were also given the option to answer “not relevant to me”. The number of GPs providing a score varied from 937 (school health service) to 1,304 (recent everyday symptoms and complaints).

For each task we calculated the proportion of GPs who scored 4 or 5 on the meaningfulness scale. With respect to priority, we calculated proportions that would like to spend more and less time on the different tasks. For preventive health care services, nursing home medicine and following up people certified unfit for work, *i.e.* tasks that the health authorities expect GPs to prioritize, we tested the hypotheses that the GPs’ priorities might vary by practice characteristics. We used multinominal logistic regression with GPs’ preferences for time spent on the different task, *i.e.* less time, more time or no change, as the dependent variable. Note that “no change” in this case means that the task in question was not among the three tasks each GP was allowed to select for “less time” and “more time”, respectively. Independent variables were population size of practice municipality, number of patients listed, number of GPs in the practice, specialty attainment and remuneration scheme (private practice *versus* salaried positions), and we adjusted for age and sex. We also considered using ordinal logistic regression, but regression diagnostics indicated that the proportional odds assumption was violated. We used SPSS version 19.0 for data analysis. *p*–values less than 0.05 were considered statistically significant. According to Norwegian law studies like ours do not require review by a research ethics committee. However, the study was approved by the Norwegian Social Science Data Services, which is the privacy ombudsman for all Norwegian universities as well as the Research Institute of the NMA. The funding source had no involvement in the conception and design of the study, the drafting of the manuscript or the decision to submit the article for publication.

## Results

We obtained responses from 1,308 (40%) of the GPs. The proportion of specialists in general practice was slightly higher among the respondents (66%, 95% CI 63% to 68%) compared to all Norwegian GPs (55%). Otherwise the respondents were representative of Norwegian GPs with respect to age, sex, number of patients listed and population size of practice municipality (Table 1).

In terms of meaningfulness the top ranked activities were handling common symptoms and complaints, follow up of chronic diseases, terminal care, management of risk conditions such as hypertension, hypercholesterolemia and osteoporosis, and on call emergency care (Table 2). In terms of priority, the same activities prevailed except for on call emergency health care (Table 3). More than one out of four GPs would like to spend less time on health certifications, practice administration, medically unexplained symptoms, following up persons certified unfit for work, and drug abuse/addiction (Table 3).

**Table 3:** **Proportions of GPs (n=1,308) that would like to spend more *versus *less time on common activities in general practice**^**1**^

**Activity**	**Would like to spend more time**	**Would like to spend less time**
**Follow up of chronic somatic diseases**	57%	2%
(*e.g. *COPD, heart disease, diabetes)		
**Recent everyday symptoms and complaints**	46%	3%
(*e.g. *infections, lumbago, tendinitis, head ache, dyspnea, chest pain, abdominal pain, *etc.*)		
**Follow up of chronic psychiatric diseases**	29%	6%
(*e.g. *schizophrenia, bipolar disorders, anxiety/depression)		
**Terminal care**	16%	1%
**Teaching and supervision of students and residents**	17%	2%
**Risk conditions**	22%	8%
(elevated blood pressure or cholesterol, low bone mass density)		
**Research**	14%	5%
**Meetings regarding individual patients**	12%	6%
**Nursing home medicine**	10%	6%
**Quality assurance**	13%	13%
(*e.g. *development and maintenance of guidelines/procedures)		
**Emergency health care**	12%	14%
(*e.g.* trauma/accidents, acute, serious somatic and psychiatric diseases)		
**School health service**	2%	10%
**Preventive health clinics**	2%	10%
**Meetings with local health authorities**	6%	17%
**Psychosocial problems**	7%	19%
(*e.g. *marital crises, conflicts at work)		
**Follow up of persons certified unfit for work**	8%	27%
**Drug abuse/addiction medicine**	6%	26%
**Medically unexplained symptoms**	5%	28%
(*e.g. *chronic fatigue, chronic pain syndroms)		
**Practice administration/management**	7%	30%
(*e.g. *human resource management, bookkeeping, *etc.*)		
**Health certifications**	0%	50%

^1 ^Among the activities listed, the GPs chose 3 activities they would like to spend less time on and 3 activities they would like to spend more time on.

In the mulitinominal regression analyses, smaller patient lists (< 1,200) were associated with preferences for less time on managing risk conditions (Table 4). On the other hand, GPs with larger patient lists were less likely to prefer spending time on school health services and preventive health clinics for children and adolescents (Table 4). GPs in salaried positions tended to prefer more time for school health services. Specialists in general practice, however, were less likely to want more time in school health services (Table 4). In general GPs’ preferences for time spent on preventive health care were not strongly associated with practice characteristics, and the regression models explained no more than 10% of the variability in those preferences (Table 4). Compared to older colleagues, GPs under 50 years of age were more likely to want to spend less time with those certified unfit for work (OR 1.7, CI 1.3 to 2.3). GPs with smaller patient lists (less than 1,200) were more likely to prefer more time for nursing home medicine (OR 1.8, CI 1.2 to 2.7), as were GPs working in smaller municipalities (< 20,000 inhabitants, OR 1.5, CI 1.04 to 2.31).

**Table 4:** **Multinomial logistic regression analysis of GPs’ preferences for time spent on preventive health care services**^**1**^

	**Risk conditions**		**Preventive health clinics**		**School health services**	
**Independent variables**	**Less time**	**More time**	**Less time**	**More time**	**Less time**	**More time**
	**OR (95% CI)**	**OR (95% CI)**	**OR (95% CI)**	**OR (95% CI)**	**OR (95% CI)**	**OR (95% CI)**
Age 50+	1.0 (0.6 – 1.6)	1.1 (0.8 – 1.5)	1.3 (0.8 – 2.0)	1.5 (0.6 – 3.8)	1.4 (0.9 – 2.1)	1.8 (0.6 – 5.5)
Female	1.0 (0.6 – 1.5)	1.3 (1.0 – 1.7)	0.8 (0.6 – 1.3)	1.0 (0.5 – 2.2)	**0.6 (0.4 – 0.9)**	2.0 (0.8 – 4.7)
Municipality >20,000 inhabitants	1.1 (0.7 – 1.8)	0.9 (0.7 – 1.3)	1.4 (0.9 – 2.1)	0.6 (0.2 – 1.4)	1.0 (0.7 – 1.6)	0.4 (0.1 – 1.1)
Specialist in GP	1.6 (0.9 – 2.8)	0.8 (0.5 – 1.0)	1.0 (0.6 – 1.6)	0.5 (0.2 – 1.3)	1.0 (0.6 – 1.6)	**0.2 (0.1 – 0.6)**
List size < 1,200	**1.8 (1.1 – 2.9)**	**0.7 (0.5 – 1.0)**^**2**^	**0.6 (0.4 – 0.9)**	1.4 (0.6 – 3.4)	**0.6 (0.4 – 0.9)**	2.0 (0.7 – 5.7)
< 4 GPs in the practice	**0.6 (0.3 – 0.9)**	0.8 (0.6 – 1.1)	1.4 (1.0 – 2.1)	0.7 (0.3 – 1.6)	1.0 (0.7 – 1.5)	0.6 (0.2 – 1.4)
Salaried position	1.7 (0.8 – 3.5)	1.0 (0.6 – 1.8)	0.5 (0.1 – 1.6)	1.8 (0.6 – 5.2)	0.6 (0.2 – 1.7)	**3.7 (1.5 – 9.4)**
Pseudo R-Square (Nagelkerke)	0.03		0.05		0.10	

^1 ^In the multinomial models the GPs’ preferences for using *less* time as well as *more *time were contrasted to *no change *in the time spent on different preventive health care services.

^2 ^p=0.026, CI includes 1.0 due to rounding.

## Discussion

### Principal findings

GPs reported that dealing with common symptoms and complaints, chronic diseases, risk conditions, emergencies and terminal care were the most meaningful tasks. Except for emergency health care, our respondents would also like to spend more time on these tasks. On the other hand, they would like to spend less time on health certifications, practice administration, meeting with local health authorities, medically unexplained symptoms, following up persons certified unfit for work, psychosocial problems, drug abuse and addiction medicine, preventive health clinics and school health services. With few exceptions, there were no strong associations between practice characteristics and GPs’ preferences for time spent on preventive health care, people certified unfit for work or nursing home medicine.

### Strengths and weaknesses

We are not aware of other studies regarding GPs’ views on common job tasks in terms of meaningfulness and priority. The main strength is the large sample size; although the response rate (40%) was modest, our sample encompassed 32% of all Norwegian GPs and was representative with respect to age, gender and geographical distribution. Our response rate is comparable to other online surveys, and compares favourably for surveys of busy clinicians [18]. When interpreting the results, several limitations must be borne in mind. First, our items were presented in the same order to each respondent, so we were unable to control for ordering effects. There was a statistically significant linear correlation between item order and mean scores for meaningfulness (R=-0.6, *p*=0.004), but not for priority. Second, our measures of meaningfulness and priority have not been formally tested for validity and reliability. For example, we are not able to distinguish between what GPs may prefer for themselves and what they would prefer in their patients’ best interests. To the extent that these preferences differ, our findings should be interpreted with caution. Finally, even if we were able to adjust for several important physician characteristics in our analyses of group differences, the possibility of unmeasured confounders remains. In particular, the above mentioned criticism of our survey during the data collection period, and the fact that our study was carried out during a period of heated debate about the future of general practice in Norway, may have had a negative impact on the response rate, and nature of responses, and introduced bias for which we were unable to control.

### Relation to theories and other studies

Various theoretical perspectives may be pertinent to our findings. For example, psychological theories emphasize factors such as individual needs, values, personality, self efficacy, goals, incentives and job characteristics as important for work motivation [19,20]. Economic theory assumes that individuals aim to maximise their utility (welfare, wellbeing) [21]. Empirical work indicates that for GPs, utility may depend on factors such as income [22-24], professional autonomy [14,24,25], a sense of clinical competence [12,15], and not least relationships with patients [13,14,22]. Our findings suggest that care for individual patients in terms of diagnosis and treatment of diseases is the most highly valued task among GPs, which seems consistent with both pertinent theories and previous studies.

### Implications for policy, practice and research

With the exception of patient list size, GPs’ priorities did not vary consistently by practice characteristics, specialty attainment or size of practice municipality. Proposed policy initiatives targeted at these factors *per se*, *e.g.* increasing the proportion of specialists in general practice, increasing practice size, offering more salaried positions, and merging small practices into larger ones, may not be effective in changing GPs’ priorities. Policy makers may, however, note that fairly large proportions of GPs were ready to spend less time on health certifications and practice administration. Measures to reduce administrative burdens as well as patient list size per doctor could potentially make more time available for preventive medicine, teamwork, coordination of care and other high priority tasks.

During the past decade there has been concern, particularly in the Scandinavian countries, that an increasing emphasis on risk factor management may have undesirable consequences on doctor-patient relationships, and change clinical priorities [26,27]. It has even been claimed that GPs would prefer to spend less time on risk factors such hypertension, elevated blood cholesterol and osteoporosis [28]. In contrast, we found that risk factor management was among the top ranked items, both in terms of meaningfulness and priority. In Norway GPs typically manage patients with risk factors in their own surgery, whereas they have to leave their surgery to work in preventive health clinics, school health services and nursing homes. It is conceivable that busy clinicians – *i.e.* with large patient lists – are more reluctant to leave their surgeries unless the activity is perceived as meaningful. Qualitative studies might deepen our understanding of what tasks GPs find meaningful in their work and why.

It is also noteworthy that compared to younger colleagues, GPs aged 50 and above assigned relatively higher scores (in terms of priority) to follow up of persons unfit to work. We can speculate that valuing such tasks comes with experience and long term relationships with patients, or else that medical school and/or post graduate education programs do not prepare young GPs sufficiently for these tasks. Indirectly, our findings may support initiatives for extended learning periods in community settings [29].

## Conclusions

Care for individual patients in terms of diagnosis and treatment was the most highly valued task among GPs in terms of meaningfulness and priority. If the GPs were to decide on their own, there would probably be less time spent on health certification, practice administration, meetings with local health authorities, medically unexplained symptoms, follow up of persons unfit for work, psychosocial problems, drug abuse and addiction medicine, preventive health clinics and school health services. These priorities are partly at odds with those of policy-makers and the health authorities. This suggests that in the patients’ best interests, GPs, health authorities, patient organisations and health policymakers should engage in a respectful and meaningful dialogue.

## Competing interests

The authors declare that they have no competing interests.

## Authors’ contributions

PAH designed the study, did the statistical analyses and drafted the manuscript. AE assisted with drafting and critical revision of the manuscript. OGA was responsible for data collection and participated in critical revision of the manuscript. IJA piloted the study and assisted with design and critical revision of the manuscript. ISK conceived of the study and assisted with design and critical revision of the manuscript. All authors read and approved the final manuscript.

## Pre-publication history

The pre-publication history for this paper can be accessed here:

http://www.biomedcentral.com/1471-2296/14/41/prepub
